# Establishing selection criteria for male New Zealand white rabbits in interventional radiology research using organ volumes and vessel diameters

**DOI:** 10.1186/s41747-026-00708-z

**Published:** 2026-04-28

**Authors:** Selma Saclier, Yubei He, Justus Klockner, Lasse Noack, Justus Ramtke, Julius Chapiro, Richard Ruppel, Friederike Hesse, Salma A. S. Abosabie, Juliane Katharina Unger, Robin Schmidt, Bernhard Gebauer, Lynn Jeanette Savic

**Affiliations:** 1 Department of Radiology, Charité - Universitätsmedizin Berlin, Berlin, Germany; 2 Department of Radiology and Biomedical Imaging, Yale University School of Medicine, New Haven, CT USA; 3 Experimental Clinical Research Center (ECRC) at Charité - Universitätsmedizin Berlin and Max-Delbrück-Centrum für Molekulare Medizin (MDC), Berlin, Germany; 4 Berlin Institute of Health at Charité - Universitätsmedizin Berlin, Berlin, Germany; 5 Institute of Experimental Biomedicine, University Hospital Würzburg, Würzburg, Germany; 6 Rudolf Virchow Center for Integrative and Translational Bioimaging, Julius-Maximilians-Universität Würzburg, Würzburg, Germany; 7 Taskforce Refinement C3R, Charité-Universitätsmedizin Berlin, Corporate Member of Freie Universität Berlin and Humboldt-Universitätsmedizin Berlin, Berlin, Germany; 8 Research Facilities of Experimental Medicine, Charité - Universitätsmedizin Berlin, Berlin, Germany

**Keywords:** Biometry, New Zealand white rabbits, Neoplasms (transplantation), Organ size, Radiology (interventional)

## Abstract

**Objective:**

New Zealand White (NZW) rabbits are widely used in interventional radiology research due to their suitability for human-sized treatment and imaging equipment, offering high translational potential. This study aims to define selection criteria for rabbits by correlating body weight (BW) and age with abdominal organ and vessel dimensions measured on cross-sectional imaging.

**Materials and methods:**

Computed tomography and magnetic resonance imaging scans of 80 male NZW rabbits were analyzed using 3D Slicer to measure abdominal organ volumes and vessel diameters. Additionally, an in-house nnU-Net was built for liver volumetry and validated against manual segmentations. Imaging-based measurements were confirmed by gross anatomy in five animals. Statistics included normality testing and Pearson correlation.

**Results:**

BW ranged from 2.0 to 4.5 kg (median [IQR]: 3.5 [2.9–3.8]) and age from 10.0 to 24.9 weeks (17.7 [15.0–21.4]); age correlated strongly with BW (*p* < 0.001). Organ volumes (liver, both kidneys) correlated with BW and age (all *p* < 0.001), respectively. Additionally, several vessel diameters (left common and internal iliac arteries, inferior vena cava, right common carotid artery) significantly correlated with BW and age, while the celiac trunk (*p* = 0.010), common hepatic (*p* = 0.011), and right renal artery (*p* = 0.031) correlated with BW only. The liver segmentation model achieved a Dice Similarity Coefficient of 0.91.

**Conclusion:**

BW and age in NZW rabbits correlate with both organ volumes and large vessels relevant to interventional procedures, supporting the use of biometric data as selection criteria to improve standardization, reduce complications, and enhance preclinical research quality.

**Relevance statement:**

Weight- and age-based selection of NZW rabbits improves anatomical suitability for image-guided interventions, enhancing technical success and reproducibility. It may reduce complications and dropouts, avoiding false attribution of adverse events to the technique rather than biometric unsuitability.

**Key Points:**

- Lack of standardized selection criteria in the VX2 rabbit model increases procedural risks and impairs reproducibility in interventional radiology research.
- Biometric data correlate with organ and vessel dimensions, enabling estimations of anatomical suitability for image-guided procedures in interventional radiological research.
- This study establishes anatomical reference data providing a quantitative basis to standardize and refine future VX2 rabbit research.

**Graphical Abstract:**

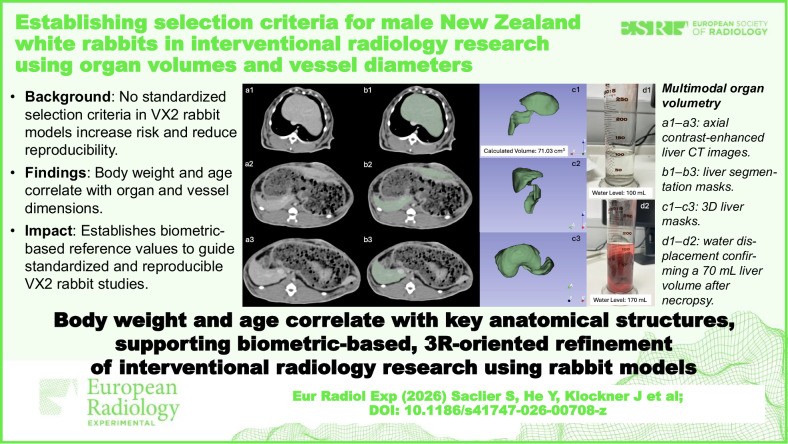

## Background

Despite continuous advances in the development of alternative methods, animal models remain indispensable for scientific and medical developments in the rapidly progressing field of biomedical research. This underscores the importance of refinement strategies that improve the scientific validity and reproducibility of preclinical models, thereby enhancing their translational potential [1].

The VX2 tumor model in New Zealand White (NZW) rabbits is the most commonly used rabbit model to study liver cancer due to its reproducibility and suitability for human-sized diagnostic and interventional equipment [2, 3]. It has been extensively applied across a broad range of interventional oncology procedures, including transarterial chemoembolization (TACE), radiofrequency and microwave ablation, irreversible electroporation, and image-guided intratumoral drug delivery [4–9]. Beyond hepatic applications, the VX2 model has been adapted to study tumor growth and treatment response in other anatomical sites such as the lung, kidney, pancreas, head, and neck region, highlighting its relevance for the preclinical evaluation of locoregional cancer therapies [10–13]. However, researchers often operate without standardized guidance for selecting appropriate animals, as many studies lack specific inclusion criteria regarding age, weight, or anatomical dimensions, and rarely report procedure-related complications. Available growth charts for NZW rabbits also present inconsistent benchmarks for body weight (BW) across age groups.

This challenge became evident in our lab when two rabbits developed limb ischemia as a serious complication after TACE. This was likely linked to individual differences in BW and vessel diameter. Previous interventions were successful using the same standardized protocol in older and thus heavier animals. Therefore, procedural errors appear unlikely to be the underlying cause, further emphasizing the role of anatomical variation. Such anatomical mismatches can result in prognostically relevant vessel injury, bleeding, or nerve irritation during femoral artery access. These observations underscore the need for precise selection criteria based on accurate vessel size measurements, both of target and access vessels, to improve procedural safety, reduce false negatives, and enable refined animal welfare-oriented methods.

This study aims to address this gap by analyzing whether BW and age correlate with abdominal organ and vessel dimensions in NZW rabbits. If such correlations exist, they may enable researchers to estimate anatomical suitability for certain interventional procedures based on easily obtainable biometric data, allowing for preprocedural selection and improved experimental consistency. Through retrospective analysis of imaging and biometric data, we aim to lay the groundwork for defining anatomical thresholds that could support safer, more standardized, and ethically appropriate animal selection. These efforts align with the 3Rs of animal research (replacement, reduction, refinement) and strengthen the translational relevance of preclinical rabbit models in interventional oncology.

## Materials and methods

### Study cohort

This multi-institutional, retrospective study was based on imaging data from animal studies approved by the institutional animal care and use committees and conducted in accordance with institutional guidelines and standards for the ethical treatment of animals.

**Fig. 1:**
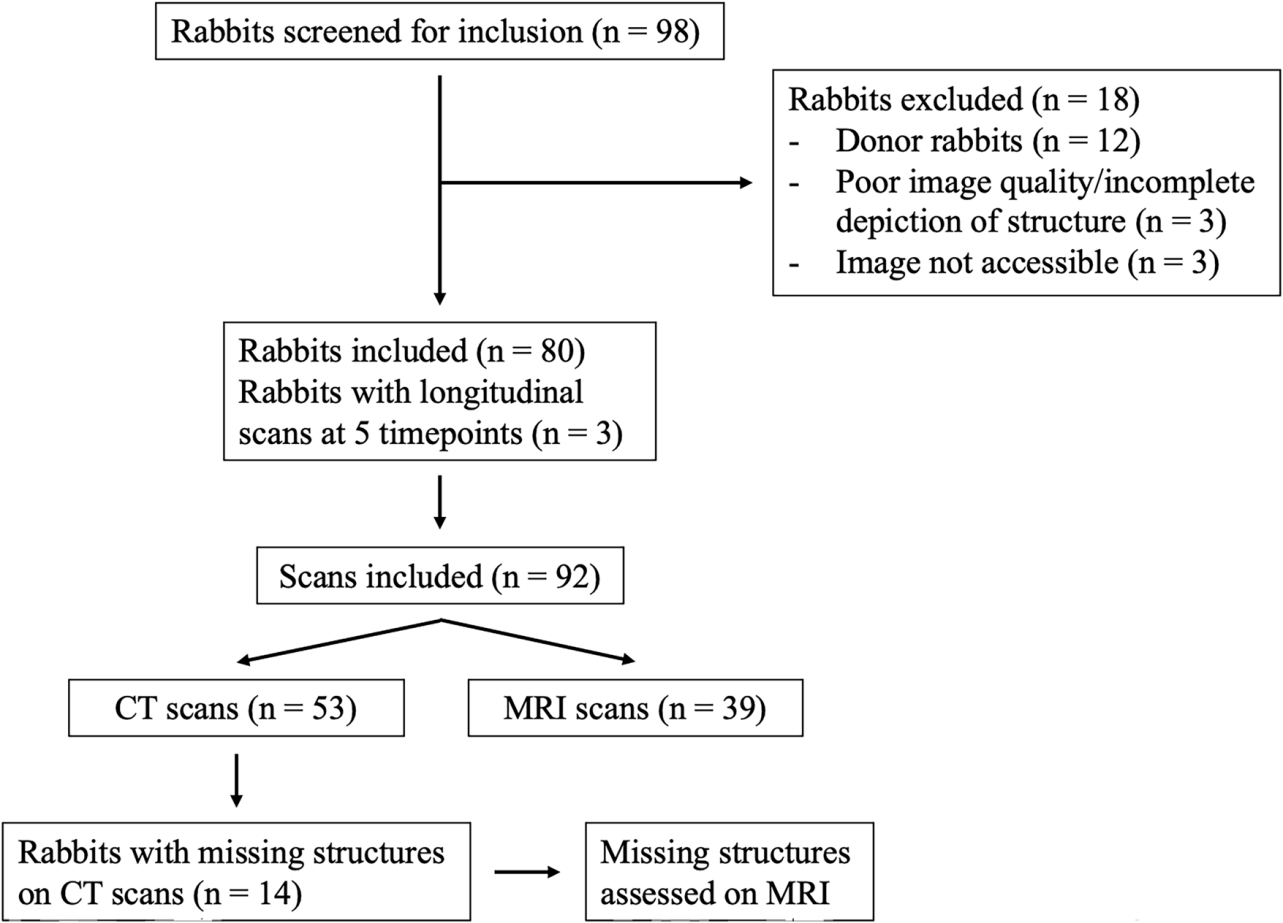
Flowchart showing the workflow of the study cohort, outlining the number of rabbits screened, included, and excluded, along with detailed reasons for exclusion. It further differentiates between CT and MRI scans and highlights the reassessment of missing anatomical structures on MRI

NZW rabbits (Charles River Laboratories Inc.) that underwent abdominal cross-sectional imaging between 2017 and 2025 were retrospectively screened. Inclusion criteria required the availability of contrast-enhanced CT or MRI scans and access to matching pre- or intraoperative documentation of the BW. Individual measurements were excluded if the corresponding imaging dataset lacked sufficient coverage, particularly when the liver, spleen, kidneys, or major abdominal vessels were not fully visualized. A total of 80 male NZW rabbits were included, with a total of 92 scans comprising 53 contrast-enhanced computed tomography (CT) and 39 magnetic resonance imaging (MRI) scans, as three rabbits underwent longitudinal imaging at five time points each (Fig. 1).

Animals were housed in laminar-flow rooms under controlled temperature and humidity. A light–dark cycle aligned with the circadian rhythm was maintained. The rabbits were kept in single cages with visual, auditory, and olfactory contact with conspecifics. They received hay and pelleted feed ad libitum. Fresh fruit and vegetables were provided daily. Drinking water was available at all times.

All animals included in this work were laboratory rabbits maintained under approved institutional animal-use protocols with predefined health monitoring procedures and humane endpoints. In line with the 3R principle (replacement, reduction, refinement), this retrospective analysis was performed using animals that were also involved in other approved experimental studies, rather than conducting additional dedicated experiments solely for the purpose of this analysis. As a result, some animals served as VX2 donor rabbits or as VX2 recipient rabbits, depending on their role within the respective study protocols. VX2 tumors were first established in donor rabbits by injection into the quadriceps muscle of the hind limb and allowed to grow for 3 weeks. Tumor tissue was then harvested, fragmented, and injected into the left hepatic lobe of recipient rabbits via median laparotomy, as described previously in the corresponding studies [14, 15]. In total, 3 donor rabbits and 77 recipient rabbits were included in our cohort.

To assess the representativeness of our cohort with respect to age and BW, focused literature research was performed in PubMed using the terms (“rabbit” OR “rabbits”) AND (“New Zealand White” OR “NZW”) AND (“VX2” OR “VX-2” OR “VX 2”) AND (“ablation” OR “embolization” OR “embolisation” OR “chemoembolization” OR “chemoembolisation” OR “TACE” OR “transarterial” OR “radioembolization”). The search was limited to publications from the last 10 years. To ensure comparability with the present experimental setup, only original research studies using the VX2 tumor model in male NZW rabbits and reporting animal age and/or weight were included, which aligns with the reporting requirements proposed by the ARRIVE guidelines [16].

### CT acquisition protocols

Contrast-enhanced CT scans in either the arterial phase (15 s post-contrast injection) or venous phase (45 s post-injection) were included for analysis. Based on the CT acquisition protocol, three cohorts can be distinguished [15, 17]:

- The first cohort (*n* = 15) was scanned using a Somatom Definition AS (Siemens Healthineers) system. Axial images were acquired with a matrix size of 512 × 512, a field of view (FOV) of 300 × 300 mm, a tube voltage of 120 kVp, a tube current of 56 mAs, and a slice thickness of 1 mm. Intravenous contrast was administered at a dose of 1 mL/kg Ultravist 350 (Bayer Healthcare).
- The second cohort (*n* = 4) was imaged on the same scanner model with identical spatial parameters and protocol settings, except for a higher tube current of 636 mAs. The contrast agent and dosage were identical to those of the first cohort.
- The third cohort (*n* = 34) underwent imaging on a LightSpeed VCT or Discovery 570c system (GE Healthcare) using a previously described triphasic liver imaging protocol [17]. Acquisition parameters included a matrix size of 512 × 512, a FOV of 219 × 219 mm, a tube voltage of 120 kVp, tube current settings of 350 mAs or 305 mAs, and a slice thickness of 0.625 mm or 0.5 mm. Contrast enhancement was achieved using 1 mL/kg of Omnipaque 350 (GE Healthcare).

### MRI acquisition protocol

Dynamic contrast-enhanced axial and coronal T1-weighted sequences were used. In cases where dynamic sequences were unavailable or incomplete, non-enhanced axial and coronal T1-weighted sequences were used. All rabbits were imaged on 3-T systems with the following specifications:

The first MRI dataset (*n* = 25) was acquired using a Biograph_mMR (Siemens Healthineers) system with a 15-channel transmit-receive knee coil. T1-weighted “volumetric interpolated breath‑hold examination” (VIBE) Dixon sequences were acquired with a 192 × 138 matrix and a slice thickness of 2.5 mm. Contrast was administered at a dose of 0.1 mmol/kg Primovist (Bayer Healthcare).

The second MRI dataset (*n* = 8) was obtained using a Magnetom Vida (Siemens Healthineers) equipped with an 18-channel transmit-receive knee coil. T1-weighted VIBE “golden‑angle radial sparse parallel”‒GRASP sequences were acquired, with a matrix size of 224 × 224 and a slice thickness of 2.5 mm. Contrast administration followed the same protocol as the first dataset.

The third MRI cohort (*n* = 6) was acquired on a Magnetom Prisma (Siemens Healthineers) using a 15-channel knee coil, following a previously described protocol [15]. Sequences included T1-weighted VIBE with controlled aliasing in parallel imaging results in higher acceleration‒CAIPIRINHA (with 2‑fold acceleration in both the phase‑encoding and the partition direction *=* 2 × 2). Parameters encompassed a matrix size of 192 × 100 and a slice thickness of 2.5 mm. Contrast-enhanced scans were performed using 0.1 mmol/kg Dotarem (Guerbet).

### Image analysis

All image analyses were performed using the open-source software 3D Slicer (version 5.6.2) [18] by two readers with one and two years of experience in abdominal imaging and preclinical imaging in rabbits, under the supervision of a third reader with 8 years of experience in the same fields. All vessel diameters were measured by a single reader, while organ volumes were evaluated independently by a second reader in addition to the first.

#### Vessel diameter measurement

Vessel diameters were measured at consistent anatomical landmarks across all animals. Arterial diameters were recorded in the arterial phase, measured immediately distal to the vessel origin. Venous diameters were recorded in the venous phase, measured proximal to their confluence with another vein. The abdominal aorta was measured 1 cm cranial to the aortic bifurcation. All vessel diameters were measured in triplicate, and the average of the three measurements was used for analysis.

#### Manual and automated organ volumetry

Only fully visualized organs were segmented. Liver, kidney, and spleen volumes were manually segmented using 3D Slicer’s (version 5.6.2) integrated tools. Liver segmentation in CT scans was supported by an in-house developed algorithm based on the nnU-Net deep learning framework [19], designed to reduce manual annotation workload and to facilitate and accelerate segmentation results. The liver segmentation model was developed using an iterative training workflow designed to reduce manual segmentation workload. An initial set of five manually delineated livers served as the starting point. Model predictions on additional cases were subsequently reviewed, corrected, and added to the training set. This cycle of prediction, manual refinement, and retraining was repeated to incrementally improve segmentation performance. All segmentation outputs were visually reviewed and manually corrected where necessary to ensure anatomical accuracy before correlation analysis.

For external validation, a separate naïve nnU-Net model was trained exclusively on the CT cohort from Yale School of Medicine (*n* = 30) and evaluated on an independent CT cohort from Charité—Universitätsmedizin Berlin (*n* = 18). This setup enabled a direct assessment of generalizability across scanners and institutions by comparing the model predictions with the corresponding manually refined liver segmentations.

Additionally, tracheal diameter was assessed 3 cm cranial to the tracheal bifurcation to obtain intubation-relevant anatomical information.

### Necropsy-based validation

**Fig. 2:**
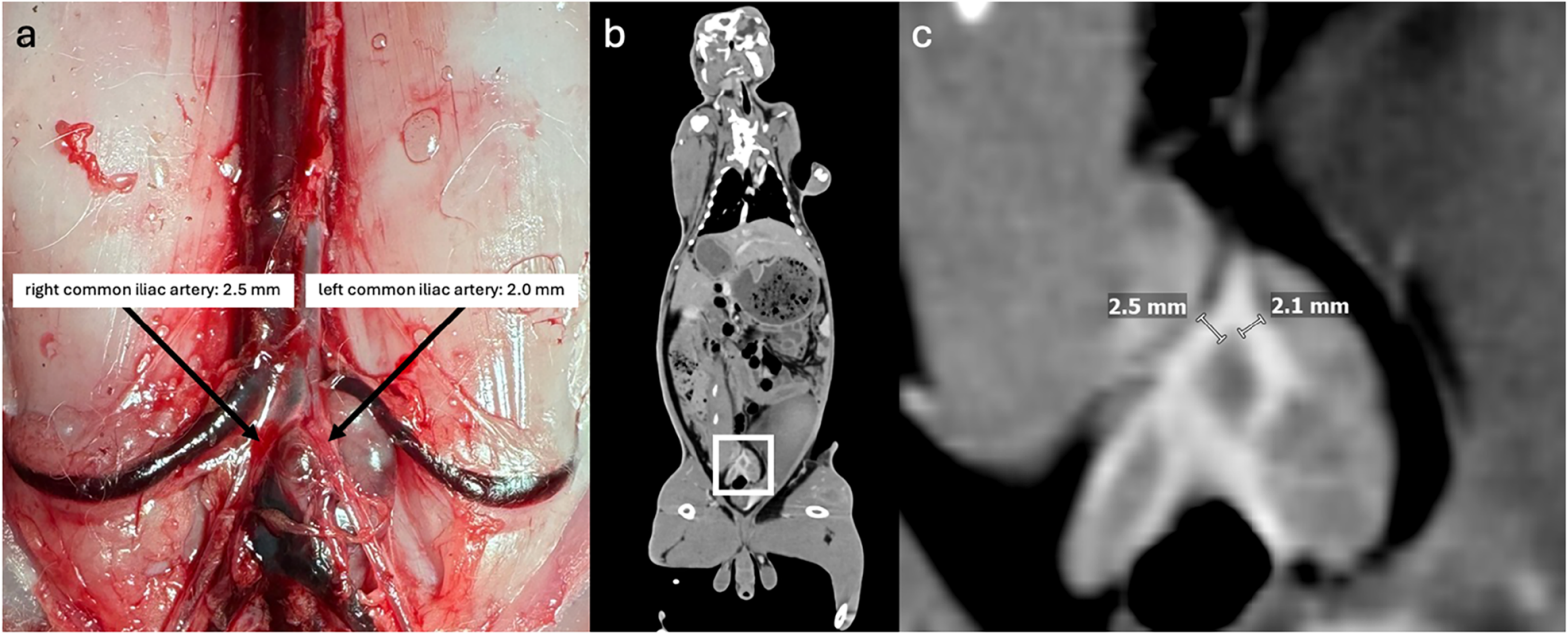
Vessel diameter measurement. **a** Common iliac arteries *in situ*. **b** Corresponding contrast-enhanced CT scan of the same rabbit in the arterial phase (15 s after contrast administration) in the coronal plane. The white frame highlights the region displayed in greater detail in **c**. All images originate from the same rabbit and illustrate that the imaging-based vessel diameter measurements could be confirmed by necropsy. These measurements may be particularly relevant for femoral access for transarterial interventions

**Fig. 3:**
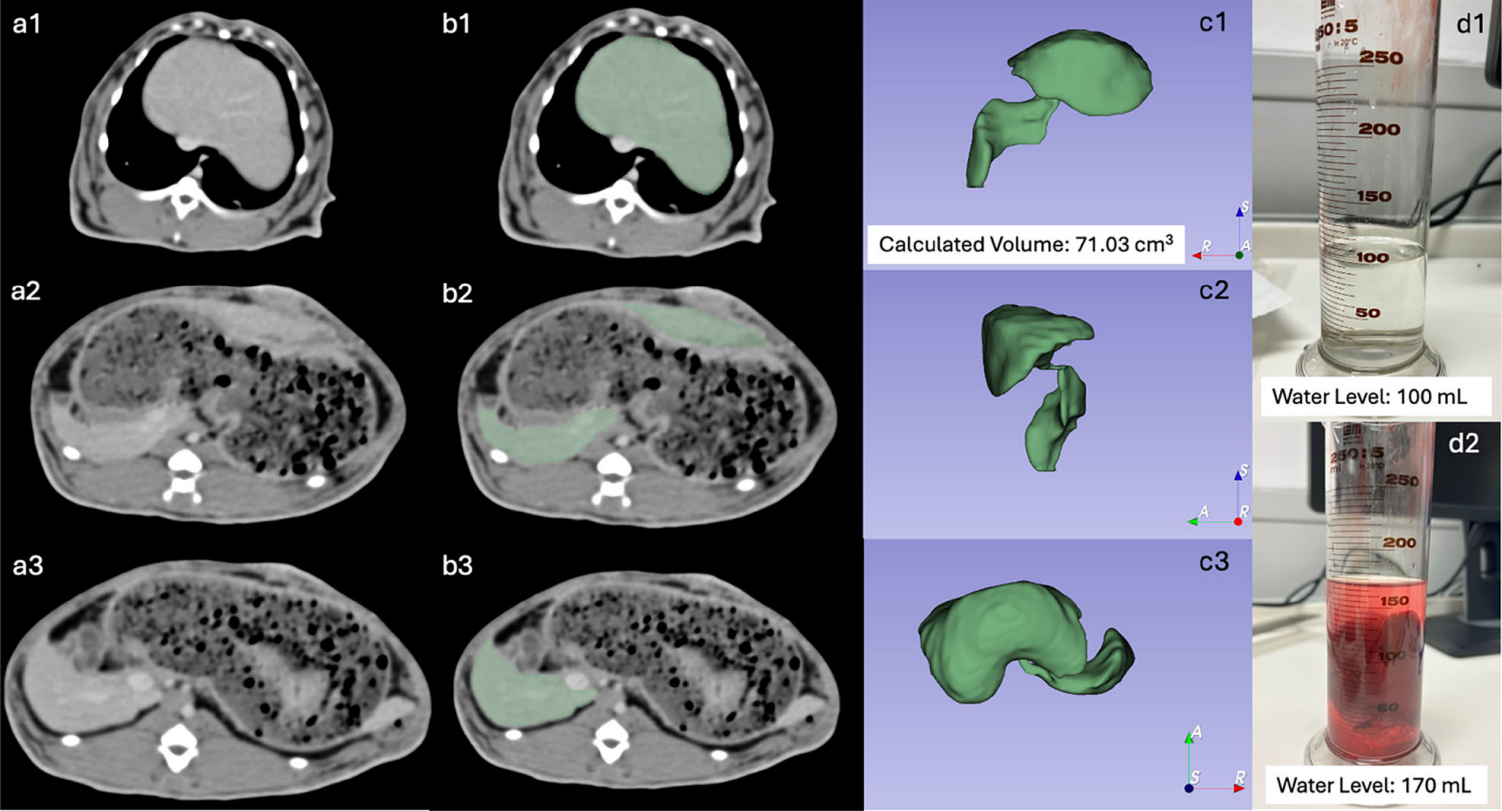
Organ volumetry. **a1**–**a3** Axial CT cross-sectional images of the liver. **b1**–**b3** Corresponding segmentation masks. **c1**–**c3** Resulting three-dimensional segmentation volume, viewed from anterior (**c1**), lateral left (**c2**), and superior (**c3**) perspectives, and calculated volume of 71.03 cm^3^. **d1** Water level of a prefilled measuring cylinder at 100 mL. **d2** Increased water level of 170 mL after immersion of the excised liver, indicating a volume of 70 mL

To validate imaging-derived anatomical measurements, necropsy was performed in five animals. In three cases, vessel diameters and organ volumes were validated; in the remaining two animals, only organ volumes were assessed. A standardized dissection protocol was developed and applied consistently across all cases to preserve anatomical integrity and ensure reproducibility. This protocol involved a stepwise approach designed to prevent premature disruption of structures intended for later measurement. Vessel diameters were measured with calipers while the vessels remained connected to the vascular system (Fig. 2). Organ volumes were determined by water displacement according to Archimedes’ principle. After detaching each organ from all vessels and adherent structures, it was immersed in a 250 mL measurement cylinder prefilled to the 100 mL mark. The organ volume was recorded as the difference between the new water level and the 100 mL baseline (Fig. 3). All results were documented in a written protocol, and photographic documentation was obtained at each stage to enable traceability and direct comparison with imaging data.

### Statistical analysis

Statistical analysis was performed using GraphPad Prism (version 10.4.1). Normality of data distributions was assessed using the Shapiro–Wilk test. Pearson correlation coefficients were calculated to evaluate associations between BW, age, and anatomical parameters, including organ volumes (liver, spleen, and kidneys) and 19 vessel diameters. According to established Cohen’s guidelines, Pearson’s correlation coefficients were interpreted as small (*r* = 0.10–0.29), medium (*r* = 0.30–0.49), or large (*r* ≥ 0.50), providing a standardized framework for assessing the strength of linear associations between variables [20]. Additionally, to analyze the growth characteristics of our study cohort in relation to reference values, an age-weight curve for all rabbits with available age data was generated. Individual BWs were plotted as scatter points, and a Gaussian kernel smoothing was applied to estimate the mean growth trajectory.

Segmentation performance of the nnU-Net-based model was evaluated using two overlap-based metrics. The Dice Similarity Coefficient was used for external validation and measures volumetric overlap between predicted and reference segmentation masks, ranging from 0 (no overlap) to 1 (perfect overlap) [21]. During cross-validation, the pseudo Dice score was natively reported by the nnU-Net framework and was used as the primary metric for internal model performance assessment [19].

To assess whether age modifies weight-to-anatomy relationships, we fitted linear regression models including an age-by-weight interaction term (Y ~ age + BW + age × BW), as previously done in interaction analyses to test for effect modification [22]. The interaction term was used to test for effect modification across the studied age range, and β estimates, 95% confidence intervals, and *p*-values are reported.

We interpreted *p*-values < 0.05 as statistically significant.

## Results

### Study cohort

The 80 male NZW rabbits included had BWs ranging from 2.0 to 4.5 kg (median [interquartile range]: 3.5 [2.9–3.8]). Of all 92 abdominal cross-sectional imaging datasets, 22 also included thoracic structures. Age information was available for 39 rabbits. Of these, three animals were scanned at five different time points each, resulting in a total of 51 age-associated data points. Recorded ages ranged from 10.0 to 24.9 weeks (17.7 [15.0–21.4]).

**Fig. 4:**
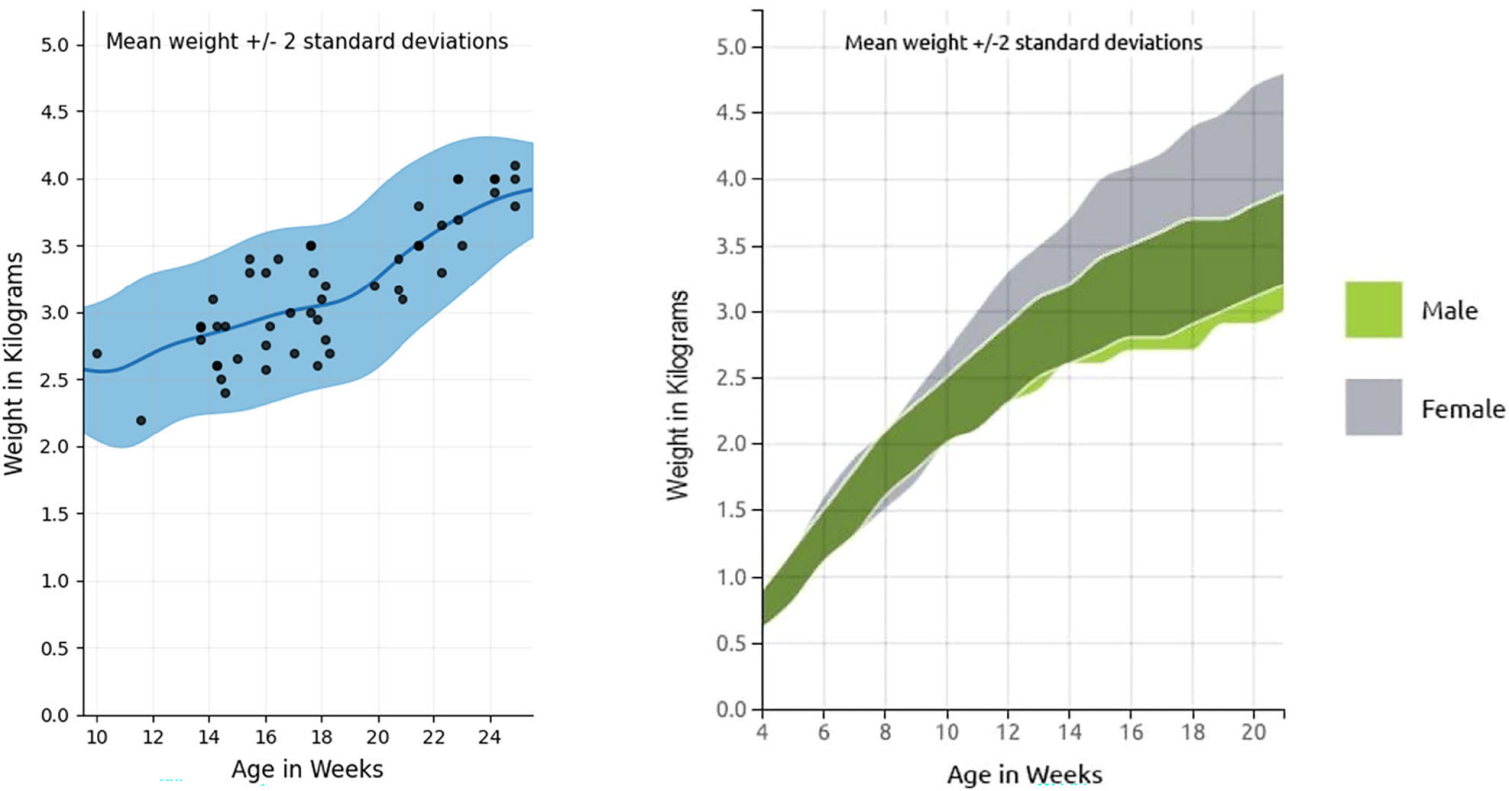
Age-weight relationship. Left: smoothed mean growth curve (blue, mean ± standard deviation shaded) with individual data points (*n* = 51) of our cohort. Right: growth chart of NZW rabbits from our breeder (Charles River). The age-weight correlation observed in our cohort (left) is consistent with the reference growth pattern provided by the breeder (right). Growth chart used by permission of Charles River Laboratories International, Inc. Growth chart data should be used as a guideline only

Significant positive and strong correlations were found between age and BW (*r* = 0.834, *p* < 0.001; *n* = 51). To validate these findings, we compared our cohort data with the reference growth chart for NZW rabbits provided by the breeder (Charles River Laboratories, Fig. 4). The observed age-weight relationship in our dataset was consistent with the reference growth pattern, thereby supporting the validity of our measurements and confirming that the animals in our study followed the expected physiological growth trajectory.

Additionally, age-by-weight interaction analyses showed no evidence of effect modification for the weight-to-anatomy relationships across the studied age range for all evaluated organ volumes and vessel diameters, except for the inferior vena cava, which demonstrated a significant interaction (*p* = 0.038) (Supplemental Table 1).

The initial PubMed search for the rapid scoping review yielded 39 publications (date of search 01/30/2026). After screening, 13 studies remained for detailed review (Supplemental Table S2).

### Correlation of vessel diameters with BW and age

**Fig. 5:**
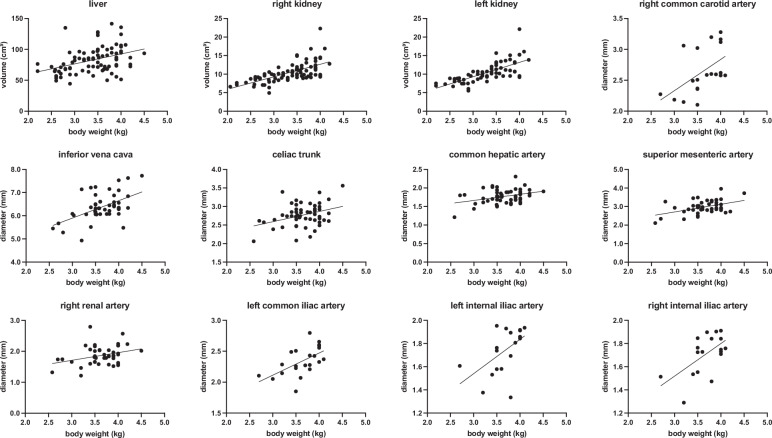
BW correlation analysis: scatter plots. This overview shows the significant (*p* < 0.05) correlations between BW and the respective parameters, including organ volumes and vessel diameter. A fitted linear regression line was added to visualize the relationship between the two variables identified by the Pearson correlation analysis

Strong significant correlations with BW were found for the right internal iliac artery (*r* = 0.586, *p* = 0.011; *n* = 18), left internal iliac artery (*r* = 0.540, *p* = 0.017; *n* = 19), left common iliac artery (*r* = 0.580, *p* = 0.005; *n* = 22), inferior vena cava (*r* = 0.522, *p* < 0.001; *n* = 50), and right common carotid artery (*r* = 0.524, *p* = 0.018; *n* = 20). Moderate correlations were observed for the superior mesenteric artery (*r* = 0.450, *p* = 0.002; *n* = 46), celiac trunk (*r* = 0.353, *p* = 0.010; *n* = 53), common hepatic artery (*r* = 0.348, *p* = 0.011; *n* = 53), and right renal artery (*r* = 0.322, *p* = 0.031; *n* = 45) (Table 1 and Fig. 5).

**Table 1:** Body weight correlation analysis

	Anatomical parameter *versus* BW	Pearson *r*	*p*-value	*n*
Volumetric measurements	Liver	0.393	**< 0.001**	86
	Right kidney	0.648	**< 0.001**	83
	Left kidney	0.698	**< 0.001**	65
	Spleen	0.070	0.641	47
Diameter measurements	Trachea	-0.130	0.585	20
	Right common carotid artery	0.524	**0.018**	20
	Left common carotid artery	0.382	0.097	20
	Superior vena cava	0.351	0.110	22
	Inferior vena cava	0.522	**< 0.001**	50
	Celiac trunk	0.353	**0.010**	53
	Common hepatic artery	0.348	**0.011**	53
	Superior mesenteric artery	0.450	**0.002**	46
	Right renal artery	0.322	**0.031**	45
	Left renal artery	0.328	0.051	36
	Abdominal aorta	0.267	0.218	23
	Right common iliac artery	0.344	0.118	22
	Left common iliac artery	0.580	**0.005**	22
	Right external iliac artery	0.383	0.079	22
	Left external iliac artery	0.385	0.077	22
	Right internal iliac artery	0.586	**0.011**	18
	Left internal iliac artery	0.540	**0.017**	19
	Right common femoral artery	-0.013	0.953	22
	Left common femoral artery	0.149	0.509	22

Pearson’s correlation coefficient was used for correlation analysis between body weight (BW) and each anatomical parameter listed. Significant (*p* < 0.05) correlations are shown in bold

**Fig. 6:**
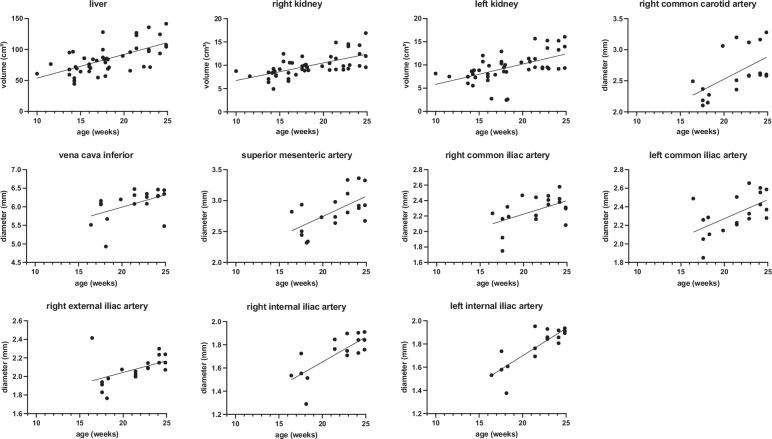
Age correlation analysis: scatter plots. This overview shows the significant (*p* < 0.05) correlations between age and the respective parameters, including organ volumes and vessel diameters. A fitted linear regression line was added to visualize the relationship between the two variables identified by the Pearson correlation analysis

Regarding age, strong correlations were found for the left internal iliac artery (*r* = 0.829, *p* < 0.001; *n* = 17), right internal iliac artery (*r* = 0.761, *p* < 0.001; *n* = 16), right common carotid artery (*r* = 0.575, *p* = 0.010; *n* = 19), superior mesenteric artery (*r* = 0.614, *p* = 0.005; *n* = 19), left common iliac artery (*r* = 0.572, *p* = 0.011; *n* = 19), right common iliac artery (*r* = 0.510, *p* = 0.026; *n* = 19), and moderate correlations with the inferior vena cava (*r* = 0.484, *p* = 0.036; *n* = 19) (Table 2 and Fig. 6).

**Table 2:** Age correlation analysis

	Anatomical parameter *versus* age	Pearson *r*	*p*-value	*n*
Volumetric measurements	Liver	0.631	**< 0.001**	47
	Right kidney	0.635	**< 0.001**	49
	Left kidney	0.559	**< 0.001**	48
	Spleen	-0.222	0.308	23
Diameter measurements	Trachea	-0.194	0.427	19
	Right common carotid artery	0.575	**0.010**	19
	Left common carotid artery	0.294	0.222	19
	Superior vena cava	0.382	0.107	19
	Inferior vena cava	0.484	**0.036**	19
	Celiac trunk	0.194	0.425	19
	Common hepatic artery	0.065	0.793	19
	Superior mesenteric artery	0.614	**0.005**	19
	Right renal artery	0.236	0.332	19
	Left renal artery	0.184	0.452	19
	Abdominal aorta	0.276	0.252	19
	Right common iliac artery	0.510	**0.026**	19
	Left common iliac artery	0.572	**0.011**	19
	Right external iliac artery	0.464	**0.045**	19
	Left external iliac artery	0.378	0.110	19
	Right internal iliac artery	0.761	**<** **0.001**	16
	Left internal iliac artery	0.829	**<** **0.001**	17
	Right common femoral artery	-0.071	0.773	19
	Left common femoral artery	0.052	0.832	19

Pearson’s correlation coefficient was used for correlation analysis between age and each anatomical parameter listed. Significant (*p* < 0.05) correlations are shown in bold

### Correlations of organ volumes with BW and age

The final in-house developed nnU-Net-based automated liver segmentation model achieved a mean pseudo Dice score of 0.97 on internal validation, and a mean Dice Similarity Coefficient of 0.91 on the external validation dataset. A strong correlation was found between BW and the left kidney volume (*r* = 0.698, *p* < 0.001; *n* = 65), right kidney volume (*r* = 0.648, *p* < 0.001; *n* = 83), and moderate correlations with the liver volume (*r* = 0.393, *p* < 0.001; *n* = 86) (Table 1 and Fig. 5).

Regarding age, strong correlations were observed for the right kidney volume (*r* = 0.635, *p* < 0.001; *n* = 49), liver volume (*r* = 0.631, *p* < 0.001; *n* = 47), and left kidney volume (*r* = 0.559, *p* < 0.001; *n* = 48) (Table 2 and Fig. 6).

## Discussion

This is the first paper to demonstrate that BW and age significantly correlate with the volumes of key abdominal organs and the diameters of several major vessels in NZW rabbits. These findings suggest that BW and age could serve as practical and non-invasive biometric surrogate markers to estimate anatomical suitability for image-guided procedures, potentially reducing procedural complications and enhancing reproducibility in interventional radiology research. Specifically, our results indicate that larger vessel calibers and organ volumes can be anticipated in heavier and or older animals, and vice versa, which may support pre-procedural planning, including selection of appropriately sized animals and matching interventional materials.

Optimizing device selection to the expected vessel caliber may reduce technical difficulty at vascular access and lower the risk of access-related complications such as vasospasm, intimal injury, dissection, or bleeding. Moreover, the correlation scatter plots and cohort-derived reference ranges reported here for the significantly correlating structures provide practical benchmarks that may facilitate animal selection and procedural preparation in future studies. Additionally, we demonstrate that automated liver segmentation using an nnU-Net can substantially reduce manual workload while maintaining strong accuracy in an external validation setting. While internal validation achieved a mean pseudo Dice score of 0.97, performance remained high on an independent external cohort from a different scanner and institution, yielding a mean Dice Similarity Coefficient of 0.91. This supports the robustness and transferability of the approach and suggests that automated liver masks can be used to streamline volumetric analyses and standardize preprocessing in future studies, with reduced dependence on time-intensive manual refinement.

As emphasized by Chang and Grieder [1], the continued relevance of animal models in biomedical research hinges not only on their translational validity but also on efforts to minimize variability and enhance procedural robustness. The significant correlations observed in our study provide a quantitative basis for such refinement. As prior studies using the VX2 model have focused primarily on treatment efficacy and technical feasibility, none have systematically investigated anatomical variability of a range of vessels and organ volumes, nor proposed selection criteria to improve preprocedural planning. By establishing weight- and age-based selection criteria, our work complements these efforts by improving model consistency from the outset.

Tam et al [23] characterized the hepatic arterial anatomy of 222 male NZW rabbits with BWs ranging between 2.7 and 4.7 kg (mean: 3.7) using hepatic arterial angiograms, identifying several branching variants of the hepatic artery with direct implications for interventional study design. While they reported no significant correlation between BW and the diameter of major hepatic arteries, including the common hepatic artery, proper hepatic artery, right hepatic artery, and left hepatic artery, this analysis was not the primary focus of their investigation, which primarily aimed to describe the normal anatomy and anatomic variations of the celiac axis and hepatic arteries in the rabbit. In contrast, our study was specifically designed to evaluate biometric predictors of anatomical suitability and demonstrated moderate yet statistically significant correlations between BW and the diameter of the common hepatic artery (*r* = 0.348, *p* = 0.011), as well as the celiac trunk (*r* = 0.353, *p* = 0.010). These differences may be attributable to variations in imaging modality (angiography *versus* cross-sectional imaging), anatomical landmarks for measurement, or animal characteristics (*e.g.*, broader range of BWs in our cohort). Based on male NZW rabbits, our results suggest that BW and age can serve as pragmatic surrogate markers to anticipate anatomical suitability for catheter-based procedures, especially when combined with imaging-based preprocedural planning.

Male NZW rabbits are reported by the breeder to reach sexual maturity at about 17–26 weeks. As animals in our cohort ranged from 10.0 to 24.9 weeks, a considerable proportion were likely prepubertal. To assess potential developmental effects, we performed an age-by-BW interaction analysis (Y ~ age + BW + age × BW). The interaction term was non-significant for all evaluated organ volumes and vessel diameters except for the inferior vena cava, indicating no meaningful age-dependent modification for most measures (Supplemental Table S1). These results should be interpreted considering the restricted availability of age data. Importantly, the aim of this study was not to investigate hormonal growth mechanisms but to provide pragmatic selection guidance under real-world experimental conditions. In this context, our review of the VX2 literature shows that most studies include rabbits within similar age and weight ranges, often with heterogeneous age distributions and frequent inclusion of pre- or peripubertal animals, as summarized in Supplemental Table S2.

When analyzing the correlation between BW and age, our findings align well with the growth chart provided by the breeder, confirming the proposed weight estimates per age (Fig. 4). In this regard, only male rabbits were included in this study, as the imaging data were derived from prior experimental studies that exclusively enrolled male animals. This approach is consistent with the majority of published VX2 tumor studies [5, 7, 17], which predominantly include male rabbits. Notably, the breeder’s reference chart indicates that female rabbits are expected to reach higher BWs than males at the same age. Given that our study demonstrates significant correlations between BW and several procedurally relevant structures, it may be worth discussing whether female rabbits could potentially be more suitable for interventional procedures. Because our cohort consisted exclusively of male rabbits, assessing sex-related differences lies outside the scope of this study.

Nevertheless, several limitations should be considered. First, the retrospective nature and heterogeneity in imaging modalities, scanners, and protocols may introduce measurement variability. Although consistent segmentation protocols were used and imaging-derived measurements were validated against anatomical dissection, residual acquisition-related variability cannot be excluded and may have attenuated observed correlations. Second, the small caliber of the examined vessels makes precise quantification inherently challenging. Minor deviations are unavoidable for millimeter-scale measurements; therefore, all values were derived from the mean of three independent measurements. Because subtle diameter variations along the vessel course cannot be reliably detected with imaging-based methods, measurements were consistently obtained at predefined anatomical landmarks to ensure reproducibility. Third, matched imaging and necropsy data were available only in a small subset due to the prospective nature of this sub-study, which may limit statistical robustness. However, this subset was included for targeted technical validation rather than anatomical characterization, and imaging-derived and *ex vivo* measurements showed high concordance without systematic bias, supporting the validity of the measurement pipeline. Finally, age information was available for only 39 of 80 rabbits, all from the more recent cohort at Charité—Universitätsmedizin Berlin, which limits statistical power for age-related analyses compared with BW-based assessments.

In conclusion, our results support the implementation of weight- and age-based selection criteria for male NZW rabbits in preclinical studies of interventional radiology. This approach may improve the reproducibility of catheter-based procedures, reduce complication rates by avoiding anatomical mismatches, and thereby contribute to the refinement and ethical optimization of preclinical study designs. Such efforts not only align with the 3Rs (replacement, reduction, refinement) but also reinforce the translational relevance of animal research in interventional oncology.

## Supplementary information

**Table S1** Sensitivity analysis. **Table S2** Characteristics of New Zealand White rabbits in interventional oncology research. Rapid scoping review of male New Zealand White rabbits included in interventional oncology studies reporting on age and/or weight published within the last 10 years (01/2016 to 01/2026)

## Data Availability

The datasets generated and analyzed during the current study are not publicly available but are available from the corresponding author upon reasonable request.

## Abbreviations

BW: Body weight
CT: Computed tomography
MRI: Magnetic resonance imaging
NZW: New Zealand White
TACE: Transarterial chemoembolization
VIBE: Volumetric interpolated breath‑hold examination
