# Supplementary material for: Establishing selection criteria for male New Zealand white rabbits in interventional radiology research using organ volumes and vessel diameters
Source: Eur Radiol Exp. 2026 Apr 28;10:57. doi: 10.1186/s41747-026-00708-z (PMC13125672; doi:10.1186/s41747-026-00708-z)
Supplement: Supplementary file 1 — Table S1 Sensitivity analysis. Table S2 Characteristics of New Zealand White rabbits in interventional oncology research. Rapid scoping review of male New Zealand White rabbits included in interventional oncology studies reporting on age and/or weight published within the last 10 years (01/2016 to 01/2026) [file 41747_2026_708_MOESM1_ESM.pdf]

Establishing selection criteria for male New Zealand White rabbits in interventional radiology research using organ volumes and vessel diameters

ELECTRONIC SUPPLEMENTARY MATERIAL

Table S1 Sensitivity analysis

Parameter	*n*	β (age × weight)	95% confidence interval	*p*-value (age × weight)
Liver	47	0.640	[-2.077 ; 3.357]	0.637
Right kidney	49	0.045	[-0.242 ; 0.333]	0.753
Left kidney	48	0.247	[-0.150 ; 0.653]	0.228
Spleen	23	-0.112	[-0.348 ; 0.124]	0.333
Trachea	19	0.073	[-0.378 ; 0.524]	0.735
Right common carotid artery	19	-0.035	[-0.278 ; 0.209]	0.766
Left common carotid artery	19	< 0.001	[-0.280 ; 0.280]	0.998
Superior vena cava	19	-0.082	[-0.487 ; 0.322]	0.671
Inferior vena cava	19	-0.267	[-0.519 ; -0.016]	0.038*
Celiac trunk	19	-0.047	[-0.281 ; 0.188]	0.676
Common hepatic artery	19	0.043	[-0.117 ; 0.203]	0.577
Superior mesenteric artery	19	-0.091	[-0.287 ; 0.105]	0.338
Right renal artery	19	0.072	[-0.179 ; 0.322]	0.551
Left renal artery	19	0.061	[-0.101 ; 0.223]	0.435
Abdominal aorta	19	0.055	[-0.143 ; 0.252]	0.564
Right common iliac artery	19	-0.062	[-0.199 ; 0.076]	0.354
Left common iliac artery	19	0.044	[-0.088 ; 0.176]	0.485
Right external iliac artery	19	0.036	[-0.074 ; 0.147]	0.491
Left external iliac artery	19	0.029	[-0.104 ; 0.163]	0.646
Right internal iliac artery	16	-0.090	[-0.182 ; 0.001]	0.053
Left internal iliac artery	17	-0.046	[-0.129 ; 0.036]	0.243
Right femoral artery	19	0.065	[-0.102 ; 0.233]	0.417
Left femoral artery	19	0.068	[-0.082 ; 0.217]	0.350

Interaction models (Y ~ age + body weight + age×body weight) were used to assess whether age modifies weight-to-anatomy relationships. Significant *p*-values are indicated by an asterisk (*).

Table S2 Characteristics of New Zealand White rabbits in interventional oncology research. Rapid scoping review of male New Zealand White rabbits included in interventional oncology studies reporting on age and/or weight published within the last 10 years (01/2016 to 01/2026)

Reference	Tumor model	*n* (rabbits)	Sex	Body weight	Age
Elkhadragy et al. [24]	VX2 liver	16	male	2.7 – 3.2 kg	
Berz et al. [25]	VX2 liver	24	male	2.5 – 4 kg	
Cheng et al. [26]	VX2 liver	16	male	2.0 – 2.5 kg	8 weeks
Thompson et al. [27]	VX2 flanks	16	male	≈ 3 kg	
Shi et al. [28]	VX2 liver	36	male	2.0 – 2.5 kg	
Kim et al. [29]	VX2 liver	20	male	2.9 – 3.4 kg	
Borde et al. [30]	VX2 liver	12	male	4.3 ± 0.2 kg	
Savic et al. [15]	VX2 liver	21	male	2.5 – 4 kg	11 – 17 weeks
Savic et al. [17]	VX2 liver	32	male	2.4 – 4 kg	
Zhang et al. [31]	VX2 liver	10	male	2 – 2.5 kg	3 – 4 months (≈ 13 – 17 weeks)
Luo et al. [32]	VX2 sciatic nerve	26	male	2.0 – 2.5 kg	
Ludwig et al. [33]	VX2 liver	10	male	3.3 – 4.19 kg	
Attaluri et al. [34]	VX2 liver	10	male	4.0 – 4.5 kg	

24 Elkhadragy L, Khabbaz RC, Muchiri RN et al (2022) Pharmacokinetics and Early Tumor Response to Conventional Transarterial Chemoembolization with Sorafenib and Doxorubicin in a VX2 Rabbit Tumor Model. J Vasc Interv Radiol 33:1213-1221 e1215. https://doi.org/10.1016/j.jvir.2022.07.011

25 Berz AM, Santana JG, Iseke S et al (2022) Impact of Chemoembolic Regimen on Immune Cell Recruitment and Immune Checkpoint Marker Expression following Transcatheter Arterial Chemoembolization in a VX2 Rabbit Liver Tumor Model. J Vasc Interv Radiol 33:764-774 e764. https://doi.org/10.1016/j.jvir.2022.03.026

26 Cheng Z, Qin H, Cao W et al (2023) Intravoxel incoherent motion imaging used to assess tumor microvascular changes after transarterial chemoembolization in a rabbit VX2 liver tumor model. Front Oncol 13:1114406. https://doi.org/10.3389/fonc.2023.1114406

27 Thompson EA, Fowlkes NW, Jacobsen MC, Layman RR, Cressman ENK (2023) Quantitative Dual-Energy CT Image Guidance for Thermochemical Ablation: In Vivo Results in the Rabbit VX2 Model. J Vasc Interv Radiol 34:782-789. https://doi.org/10.1016/j.jvir.2022.12.026

28 Shi Q, Li T, Huang S et al (2021) Transcatheter Arterial Embolization Containing Donafenib Induces Anti-Angiogenesis and Tumoricidal CD8(+) T-Cell Infiltration in Rabbit VX2 Liver Tumor. Cancer Manag Res 13:6943-6952. https://doi.org/10.2147/CMAR.S328294

29 Kim GM, Kim MD, Kim do Y et al (2016) Transarterial Chemoembolization Using Sorafenib in a Rabbit VX2 Liver Tumor Model: Pharmacokinetics and Antitumor Effect. J Vasc Interv Radiol 27:1086-1092. https://doi.org/10.1016/j.jvir.2016.02.032

30 Borde T, Laage Gaupp F, Geschwind JF et al (2020) Idarubicin-Loaded ONCOZENE Drug-Eluting Bead Chemoembolization in a Rabbit Liver Tumor Model: Investigating Safety, Therapeutic Efficacy, and Effects on Tumor Microenvironment. J Vasc Interv Radiol 31:1706-1716 e1701. https://doi.org/10.1016/j.jvir.2020.04.010

31 Zhang L, Wang N, Mao J et al (2018) Dual-Energy CT-Derived Volumetric Iodine Concentration for the Assessment of Therapeutic Response after Microwave Ablation in a Rabbit Model with Intrahepatic VX2 Tumor. J Vasc Interv Radiol 29:1455-1461. https://doi.org/10.1016/j.jvir.2018.04.019

32 Luo X, Qin Z, Tao H et al (2017) The Safety of Irreversible Electroporation on Nerves Adjacent to Treated Tumors. World Neurosurg 108:642-649. https://doi.org/10.1016/j.wneu.2017.09.049

33 Ludwig JM, Xing M, Gai Y, Sun L, Zeng D, Kim HS (2017) Targeted Yttrium 89-Doxorubicin Drug-Eluting Bead-A Safety and Feasibility Pilot Study in a Rabbit Liver Cancer Model. Mol Pharm 14:2824-2830. https://doi.org/10.1021/acs.molpharmaceut.7b00336

34 Attaluri A, Seshadri M, Mirpour S et al (2016) Image-guided thermal therapy with a dual-contrast magnetic nanoparticle formulation: A feasibility study. Int J Hyperthermia 32:543-557. https://doi.org/10.3109/02656736.2016.1159737
